# Total ankle replacement versus ankle fusion for end-stage ankle osteoarthritis: a narrative review of the latest literature data (2023–2025)

**DOI:** 10.1530/EOR-2025-0106

**Published:** 2026-08-03

**Authors:** E Carlos Rodríguez-Merchán, Inmaculada Moracia-Ochagavía, Alberto D Delgado-Martínez

**Affiliations:** ^1^ Department of Orthopaedic Surgery, Hospital Universitario La Paz, Madrid, Spain; ^2^ Osteoarticular Surgery Research, Hospital La Paz Institute for Health Research – IdiPAZ (Hospital Universitario La Paz – Universidad Autónoma de Madrid), Madrid, Spain; ^3^ Department of Orthopaedic Surgery, Hospital Universitario de Jaén, Jaén, Spain; ^4^ Department of Surgery, University of Jaén, Jaén, Spain

**Keywords:** ankle, osteoarthritis, total ankle replacement, ankle fusion, comparative studies

## Abstract

- This review compares the outcomes of total ankle replacement (TAR) and ankle fusion (AF) in patients with end-stage ankle osteoarthritis (OA) based on recent comparative studies (1 January 2023 to 31 December 2025).
- Racial/ethnic, socioeconomic, and payer status disparities have been reported in the likelihood of experiencing TAR versus AF for ankle OA.
- A prospective multicentre study with level II evidence found that the long-term clinical results of TAR and AF were similar. A meta-analysis identified TAR as the superior intervention, demonstrating significant advantages in patient-reported outcome measures.
- Three systematic reviews have reached the following conclusions: i) postoperative outcomes are similar in TAR and AF; ii) TAR and AF have similar complication rates (both minor and major); iii) both TAR and AF are safe and effective surgical treatments for ankle OA; besides, TAR had notable lower total complications, implant removals, adjacent level fusion surgeries, and non-union/open reduction and internal fixation surgeries after the index procedure.
- The existence of mixed evidence found in the literature makes it necessary to select the surgical technique to be used (TAR or AF) on an individual basis.
- To definitively determine which procedure is more appropriate in advanced ankle OA, more and better-designed studies are required, given that the results reported thus far do not permit to determine with absolute certainty which of the two procedures, TAR or AF, is more adequate.

## Introduction

Standard treatment methods for end-stage ankle osteoarthritis (OA) include ankle fusion (AF) and total ankle replacement (TAR) (1, 2, 3).

In 2022, Watts *et al.* published a systematic review of the period 2006–2019 in which they compared TAR and AF (tibiotalar fusion) (4). Individual cohort studies and randomised controlled trials were included in the study. Results were assessed at 2 and 5 years. The primary outcomes were patient-reported outcome measures (PROMs), revision and re-operation rates, and complications. No significant difference in PROMs was evident at 2 years post-surgery (American Orthopaedic Foot and Ankle Society (AOFAS) scores were 78.8 and 80.8 for the TAR and AF groups, respectively). At 2 years post-surgery, the revision rates for the TAR and AF groups were similar (3.5 and 3.7%, respectively); however, the re-operation rate was 2.5 times higher for the TAR cohort (12.2%) in comparison with the AF cohort (5.1%). Documented complications in the TAR cohort were half those documented in the AF cohort 2 years post-surgery. No AF studies were encountered that contained mean 5-year follow-up information within the study period. The conclusion was that despite new developments in TAR design, no clear evidence was found of their superiority over AF when considering patient outcomes 2 years postoperatively. However, Watts *et al.* also stated that their conclusion could be debatable in certain individuals, such as diabetic patients and patients with stiff hindfoot and midfoot (4).

For this reason, it was decided to conduct a new literature review for the period 1 January 2023–31 December 2025. This narrative review compares the outcomes of TAR and AF based on recent comparative studies.

Table 1 shows how the literature search was conducted in PubMed using four sets of keywords. Of the 107 articles found, 20 were included in the reference list because they were actually comparative studies of the two techniques (13 articles) containing highly relevant information on TAR and AF (13 articles) or non-comparative studies that also contained highly relevant information on TAR and AF. The remaining 87 were excluded because they were not comparative studies and because their information was considered to be of little interest with regard to the objective of this article.

**Table 1:** Publications on TAR versus AF from 1 January 2023 to 31 December 2025. Literature search was conducted in PubMed using four sets of keywords.

Keywords	2023	2024	2025	Total articles
TAR vs AA	17 articles	7 articles	8 articles	32
TAR vs AF	13 articles	4 articles	3 articles	20
TAA vs AA	16 articles	7 articles	11 articles	34
TAA vs AF	13 articles	4 articles	4 articles	21
Total				107*

* Only 20 were analysed; 13 of them compared TAR versus AF and are included in Tables 2 and 3.

TAR, total ankle replacement; AA, ankle arthrodesis; TAA, total ankle arthroplasty; AF, ankle fusion.

## Comparative studies: TAR versus AF

Schmerler *et al.* (5) found that black and Asian individuals were 34 and 41%, respectively, less likely than white individuals to experience TAR rather than AF. Individuals in income quartiles 3 and 4 were 22 and 32% more likely, respectively, than individuals in quartile 1 to experience TAR rather than AF. In individuals <65 years of age, privately insured and Medicare individuals were 84 and 37% more likely, respectively, than Medicaid individuals to experience TAR rather than AF. Racial/ethnic, socioeconomic, and payer status disparities existed in the likelihood of experiencing TAR versus AF for ankle OA (5).

In a comparison of loss of bone height after TAR versus AF (tibiotalocalcaneal arthrodesis), Vesely *et al.* (6) found a loss of height in the AF cohort and an increased anterior and posterior height in the TAR cohort. However, when comparing the TAR and AF cohorts, only the anterior height measurement reached statistical significance when stratified by gender. The potential change in height appeared to be an important factor to take into account during surgical planning, as a limb length discrepancy might result (6).

### Outcome measurements

#### PROMs

Goldberg *et al.* (7) were unable to demonstrate a statistically significant difference between TAR and AF based on the change in the Manchester–Oxford Foot Questionnaire walking/standing domain scores between the preoperative baseline and 4.3 years post-surgery. However, a post hoc analysis of fixed-bearing TAR showed a statistically significant improvement over AF in the Manchester–Oxford Foot Questionnaire walking/standing domain score (7).

The results of Almutairi *et al.* (8) showed a significantly larger increase in the overall range of motion (ROM) in TAR than in AF (8). In 2024, Li *et al.* stated that in the short-term, TAR demonstrated better clinical scores (Foot and Ankle Ability Measure (FAAM) scores) than AF. However, in the long term, AF exhibited superior clinical scores (9). In a study by Glazebrook *et al.* (10), TAR and AF demonstrated similar functional results using the Ankle Osteoarthritis Scale (10). In 2025, Liu *et al.* identified TAR as the superior surgical procedure, demonstrating significant advantages in AOFAS scores, FAAM activities of daily living, FAAM total scores, and SF-36 (the 36-Item Short Form Health Survey) total scores (11).

In 2024, Rodriguez-Merchan and Moracia-Ochagavía published a narrative review of the literature in which they analysed the comparative outcomes (TAR versus AF) of articles published in 2021 and 2022 in patients with ankle OA. Regarding PROMs, no differences were found between TAR and AF, although both surgical procedures improved PROMs with respect to the preoperative situation. That is, both procedures (AF and TAR) were effective in terms of improving preoperative symptoms due to advanced ankle OA (12).

#### Revision and re-operation rates

In a meta-analysis that compared the outcomes of TAR and AF based on comparative studies, Liu *et al.* (11) found a significant difference in revision rates favouring AF (11). In the Glazebrook *et al.* (10) study, individuals in the AF cohort had a greater chance of requiring no further surgery after their index procedure compared with those in the TAR cohort (70 vs 58%) (10).

In a meta-analysis of five studies (two RCTs (randomised clinical trials) and three score-matched studies) totalling 13,957 patients (6,975 TARs; 6,982 AFs), Boey *et al.* (2025) found that revision surgery or implant removal was the most frequently occurring major complication in both TAR and AF (13).

#### Complications

Ross *et al.* (14) found that individuals who experienced AF exhibited significantly higher rates of at least one common joint complication at 90 days (19.3 versus 12.6%), 1 year (25.6 versus 15%), and 2 years (26.9 versus 16.2%) postoperatively. This included higher rates of adjacent fusion or osteotomy procedures, periprosthetic fractures, and hardware removal at each postoperative follow-up. Rates of prosthetic joint infection were comparable at 2 years postoperatively (4.3 versus 4.2%) (14).

The results of Almutairi *et al.* (8) showed a significantly lower risk of infections in TAR than in AF (8). In a study by Anigian *et al.* (15), there were no differences between TAR and AF when comparing superficial infection, wound dehiscence, or readmissions in the first 30 days (15). In 2024, Li *et al.* stated that in the short term, TAR demonstrated lower complication rates. Conversely, in the long term, AF exhibited lower complication rates, although this difference was not statistically significant (9).

In 2024, Rodriguez-Merchan and Moracia-Ochagavía found that complication rates at 3 months, 1 year, and 2 years were higher after AF than after TAR (13, 15, and 16%, respectively). However, infection rates were similar (about 4%). The complication rate after AF was 25 versus 15% after TAR, and the revision rate was 16% after AF versus 11% after TAR (12).

In a meta-analysis of five studies (two RCTs (randomised clinical trials) and three score-matched studies) totalling 13,957 patients (6,975 TARs; 6,982 AFs), Boey *et al.* (2025) found that the pooled relative risk (RR) for total complications was 0.95 (95% CI: 0.85–1.08, *P* = 0.45, moderate certainty, favourable towards AF). The pooled RR for major complications was 1.18 (95% CI: 0.70–1.98; *P* = 0.54, low certainty, favouring TAR), and for minor complications, it was 0.96 (95% CI: 0.75–1.22; *P* = 0.73, moderate certainty, favouring AF). Wound dehiscence or complications, not including deep infections, were the most frequently occurring minor complication in both TAR and AF (13).

#### Cost-effectiveness

In a pragmatic, multicentre, parallel-group, non-blinded randomised controlled trial, individuals with end-stage ankle OA aged 50–85 years and considered appropriate for both procedures were recruited from 17 hospitals in the United Kingdom and randomised using minimisation (Goldberg *et al.* (7)). It was estimated that there was a 69% probability that TAR was more cost-effective compared with AF at the National Institute for Health and Care Excellence’s cost-effectiveness threshold of £20,000 per quality-adjusted life-year gained over the patient’s lifetime (7).

Silvestre *et al.* (16) identified several healthcare consumer characteristics associated with TAR preference, including male sex, higher level of education, current or previous position in health care, and history of lower extremity surgery. Conversely, increased household income and a history of moderate exercise were associated with AF preference (16).

In 2025, Encinas *et al.* stated that both TAR and AF were safe and effective surgical treatments for ankle OA. TAR had notable lower total complications, implant removals, adjacent level fusion surgeries, and non-union/open reduction and internal fixation surgeries after the index procedure (17).

Table 2 presents detailed information from articles with level II and level III evidence on comparative results of TAR versus AF (8, 9, 10, 11, 13, 14, 19). Table 3 presents detailed information from articles with level IV evidence on comparative results of TAR versus AF (5, 6, 7, 12, 15, 16).

**Table 2:** Total ankle replacement (TAR) versus ankle fusion (AF) for end-stage ankle osteoarthritis (OA): seven studies with level II and level III evidence (1 January 2023–31 December 2025).

Study	Year	Methods	Results	Conclusions
Ross *et al.* (14)	2023	Level III (retrospective study)	AF showed higher rates of at least one common joint complication at 2 years (26.9 vs 16.2%). Rates of PJI were comparable at 2 years (4.3 vs 4.2%)	AF cohort exhibited higher rates of postoperative joint complications (adjacent fusion or osteotomy procedures, periprosthetic fractures, and hardware removal) at 3 months
Almutairi *et al.* (8)	2023	Systematic review and meta-analysis	A significantly lower risk of infections in TAR than in AF, amputations, and postoperative non-union, and a significant increase in overall ROM in TAR than in AF	Rates of infection, amputation, and postoperative non-union were inferior in TAR than in AF
Li *et al.* (9)	2024	Systematic review	No significant difference in postoperative SF-36 scores between the TAR and AF groups. However, the postoperative FAAM scores in the AF group were significantly higher than in the TAR group	In the short term, TAR demonstrated better clinical scores than AF and lower complication rates. Conversely, in the long-term, no statistical difference was found
Boey *et al.* (13)	2025	Systematic review and meta-analysis	Pooled RR for total complications was 0.95, favourable towards AF	Pooled RR for major complications was 1.18, favouring TAR, and for minor complications was 0.96, favouring AF
Glazebrook *et al.* (10)	2025	Level II	Patients (*n* = 211) in the AF group had a greater chance of requiring no further surgery following their index procedure than those in the TAR group (70 vs 58%). TAR and AF groups demonstrated similar functional outcomes	Long-term clinical results of TAR and AF were similar. The minimum follow-up was 10 years and the mean follow-up was 13.2 years
Liu *et al.* (11)	2025	Level III (meta-analysis)	Significant difference in the AOFAS score favouring TAR. Overall, SF-36 score reported improved outcomes for individuals who underwent TAR compared to those with AF. FAAM score also showed significant improvement in individuals who underwent TAR compared to those who underwent AF	This meta-analysis identified TAR as the superior intervention, demonstrating significant advantages in AOFAS scores and ROM. However, there was a significant difference in revision rates favouring AF
Encinas *et al.* (17)	2025	Systematic review	Lower rate for total complications (13.1 vs 31.4%), removal of implant (8.5 vs 20%), adjacent level fusion (5.1 vs 13.4%), and non-union/ORIF (1.6 vs 7.2%) was found for TAR. Minimum follow-up was 12 months	TAR had lower total complications, implant removals, adjacent level fusion surgeries, and non-union/ORIF surgeries. No notable differences were found on PROMs

PJI, periprosthetic joint infection; ROM, range of motion; SF-36, short Form-36; FAAM, Foot and Ankle Ability measure; RR, relative risk; AOFAS, American Orthopaedic Foot and Ankle Society; ORIF, open reduction and internal fixation; PROMs, patient-reported outcome measures.

**Table 3:** Total ankle replacement (TAR) versus ankle fusion (AF) for end-stage ankle osteoarthritis (OA): six studies with level IV evidence (1 January 2023–31 December 2025).

Study	Year	Methods	Results	Conclusions
Goldberg *et al.* (7)	2023	Pragmatic, multicentre, parallel-group, non-blinded RCT	At 4.3 years, the mean MOXFQ walking/standing domain score was 31.4 in TAR and 36.8 in AF. One individual in the TAR group needed revision. Rates of wound-healing issues (13.4 vs 5.7%) and nerve injuries (4.2 vs <1%) were higher, and the rate of thromboembolic events was lower (2.9 vs 4.9%) in TAR than in AF. The bone non-union rate (based on plain radiographs) in AF was 12.1%, but only 7.1% of individuals had symptoms	Both TAR and AF improved patients’ quality of life at 1 year, and both seemed to be safe. When TAR was compared with AF overall, these authors were unable to show a statistically significant difference between the two groups in terms of their primary outcome measure. The TAR versus AF trial was inconclusive in terms of the superiority of TAR
Schmerler *et al.* (5)	2023	Examination of racial/ethnic, socioeconomic, and payer status disparities	Black and Asian patients were 34 and 41% less likely, respectively, than white patients to undergo TAR rather than AF. In patients <65 years of age, privately insured and Medicare patients were 84 and 37% more likely, respectively, than Medicaid patients to undergo TAR rather than AF	Racial/ethnic, socioeconomic, and payer status disparities existed in the likelihood of undergoing TAR versus AF for ankle OA
Anigian *et al.* (15)	2023	Comparison of perioperative results after TAR and AF.	Readmission rate and LOS were similar between TAR and AF. However, AF was associated with a higher risk of perioperative complications, including deep surgical site infections and re-operations	There were no differences between the two groups in terms of superficial infection, wound dehiscence, or readmissions in the first 30 days
Rodríguez-Merchán & Moracia-Ochagavía (12)	2024	Narrative review of literature	Regarding PROMs, no differences were found between TAR and AF. That is, both procedures were effective in terms of improving preoperative symptoms due to advanced ankle OA	It appeared that TAR offered the same PROMs as AF, but with a lower rate of complications, revisions, and re-operations in some reported series
Vesely *et al.* (6)	2024	Retrospective radiographic review conducted over a 5-year period	When comparing TAR and AF, only the anterior height measurement reached statistical significance when stratified by gender	The potential change in height was an important factor to consider during surgical planning as a limb length discrepancy may result
Silvestre *et al.* (16)	2025	Analysis of consumer preferences for TAR vs AF	Respondents indicated that ankle ROM was most important, with chance of additional surgery being least important. Most respondents preferred TAR over AF (72%)	This study identified several healthcare consumer characteristics associated with TAR or AF preference

RCT, randomised controlled trial; MOXFQ, Manchester–Oxford Foot Questionnaire; LOS, length of stay; PROMs, patient-reported outcome measures.

According to Anastasio *et al.*, patients undergoing primary TAR have an average age of 64 years. Of them, 17.7% requires removal of deep implants and 10.4% requires gastrocnemius recession. From 2012 to 2020, TAR has increased in incidence throughout the United States (18).

No statistically significant differences in complication rates were observed by Comisi *et al.* between fixed-bearing (FB) and mobile-bearing (MB) TARs. Pooled complication rates revealed total event rates of 16.1% for FB implants and 18.8% for MB implants. Major complication rates were 6.8% for FB and 9.2% for MB, while minor complication rates were 9.2 and 9.6%, respectively (19).

In a single-centre cohort study published by Robert *et al.*, rheumatoid arthritis (RA) was associated with an increased risk of wound-healing complications (OR: 11.75 (95% CI: 1.06–130.32), *P* = 0.045). However, other surgical and medical complication rates were equivalent. The change in the Manchester–Oxford Foot Questionnaire (MOXFQ) score following TAR was equal between the RA and OA group after adjusting for demographic covariates (OR: 1.01 (95% CI: 0.99–10.3), *P* = 0.284) (20).

Racial/ethnic, socioeconomic, and payer status disparities have been reported in the likelihood of experiencing TAR versus AF for ankle OA (15).

A prospective multicentre study by Glazebrook *et al.* (10), with level II evidence, found that the long-term clinical results of TAR and AF were similar (10). The meta-analysis performed by Liu *et al.* (11) identified TAR as the superior intervention, demonstrating significant advantages in AOFAS scores, FAAM activities of daily living, FAAM total scores, SF-36 total scores, and ROM (10).

Currently, available evidence from a systematic review suggests no significant disparity in postoperative outcomes between TAR and AF (8). The systematic review published by Boey *et al.* found that TAR and AF have similar rates of major and minor complications (14). The systematic review published by Encinas *et al.* (17) found that both TAR and AF are safe and effective surgical treatments for ankle OA. TAR had notably lower total complications, implant removals, adjacent level fusion surgeries, and non-union/open reduction and internal fixation surgeries after the index procedure (17).

This study has two important limitations: i) it is a narrative review of the literature and ii) only articles published in PubMed have been analysed and no bibliographic searches have been conducted in other search engines.

## Conclusion

A prospective multicentre study with level II evidence encountered that the long-term clinical outcomes of TAR and AF were similar. A meta-analysis identified TAR as the superior surgical procedure, showing significant advantages in AOFAS scores, FAAM activities of daily living, FAAM total scores, SF-36 total scores, and ROM.

Three systematic reviews have reached the following conclusions: i) postoperative results were similar in TAR and AF; ii) TAR and AF had similar complication rates (both minor and major); iii) both TAR and AF were safe and effective surgical techniques for ankle OA; besides, TAR had notable lower total complications, implant removals, adjacent level fusion surgeries, and non-union/open reduction and internal fixation surgeries after the index procedure.

However, to definitively determine which procedure is more appropriate in advanced ankle OA, more and better-designed studies are required, given that the results reported thus far do not permit to determine with absolute certainty which of the two procedures, TAR or AF, is more adequate.

Therefore, the existence of mixed evidence found in the literature makes it necessary to select the surgical technique to be used (TAR or AF) on an individual basis.

## ICMJE Statement of Interest

The authors declare that there is no conflict of interest that could be perceived as prejudicing the impartiality of the research reported.

## Funding Statement

This research did not receive any specific grant from any funding agency in the public, commercial, or not-for-profit sector.

## References

[1] Syed F & Ugwuoke A. Ankle arthroplasty: a review and summary of results from joint registries and recent studies. EFORT Open Rev 2018 3 391–397. (10.1302/2058-5241.3.170029) 30034820 PMC6026887

[2] Herrera-Pérez M, Valderrabano V, Godoy-Santos AL, et al. Ankle osteoarthritis: comprehensive review and treatment algorithm proposal. EFORT Open Rev 2022 7 448–459. (10.1530/EOR-21-0117) 35900210 PMC9297055

[3] Rodríguez-Merchán EC, Ribbans WJ, Olmo-Jiménez JM, et al. Arthroscopic ankle arthrodesis for end-stage ankle osteoarthritis. EFORT Open Rev 2025 10 213–223. (10.1530/EOR-2023-0100) 40326529 PMC12061018

[4] Watts DT, Moosa A, Elahi Z, et al. Comparing the results of total ankle arthroplasty vs tibiotalar fusion (ankle arthrodesis) in patients with ankle osteoarthritis since 2006 to 2020- A systematic review. Arch Bone Joint Surg 2022 10 470–479. (10.22038/ABJS.2021.55790.2778) 35928907 PMC9295584

[5] Schmerler J, Dhanjani SA, Wenzel A, et al. Racial, socioeconomic, and payer status disparities in utilization of total ankle arthroplasty compared to ankle arthrodesis. J Foot Ankle Surg 2023 62 928–932. (10.1053/j.jfas.2023.08.004) 37595678

[6] Vesely BD, LeSavage L, Kipp J, et al. Comparison of loss of bone height following total ankle arthroplasty versus tibiotalocalcaneal arthrodesis. J Foot Ankle Surg 2024 63 136–139. (10.1053/j.jfas.2023.09.010) 37777151

[7] Goldberg AJ, Chowdhury K, Bordea E, et al. Total ankle replacement versus ankle arthrodesis for patients aged 50-85 years with end-stage ankle osteoarthritis: the TARVA RCT. Health Technol Assess 2023 27 1–80. (10.3310/PTYJ1146) PMC10150410 37022932

[8] Almutairi TA, Ragab KM, Elsayed SM, et al. Safety and efficacy of total ankle arthroplasty versus ankle arthrodesis for ankle osteoarthritis: a systematic review and meta-analysis. Foot 2023 55 101980. (10.1016/j.foot.2023.101980) 36863247

[9] Li T, Zhao L, Liu Y, et al. Total ankle replacement versus ankle fusion for end-stage ankle arthritis: a meta-analysis. J Orthop Surg 2024 32 10225536241244825. (10.1177/10225536241244825) 38607239

[10] Glazebrook M, Balasubramaniam U, Walls A, et al. Outcomes of total ankle replacement versus ankle arthrodesis for the treatment of end-stage ankle arthritis: a concise follow-up, at a minimum of 10 years, of a previous report. J Bone Joint Surg Am 2025 107 552–557. (10.2106/JBJS.24.00361) 39715299

[11] Liu J, Cho T, Arefi IA, et al. Total ankle arthroplasty versus ankle arthrodesis in end-stage osteoarthritis: a meta-analysis of comparative outcomes. J Orthop 2025 63 157–164. (10.1016/j.jor.2025.03.056) 40248053 PMC12000697

[12] Rodriguez-Merchan EC & Moracia-Ochagavia I. Results of total ankle arthroplasty versus ankle arthrodesis. Foot Ankle Clin 2024 29 27–52. (10.1016/j.fcl.2023.08.010) 38309802

[13] Boey JJE, Toh RX, Loh YC, et al. Complications of total ankle arthroplasty versus ankle arthrodesis: a systematic review and meta-analysis with trial sequential analysis. Foot Ankle Surg 2025 31 378–383. (10.1016/j.fas.2024.11.008) 39701836

[14] Ross BJ, Savage-Elliott I, Wu VJ, et al. Complications following total ankle arthroplasty versus ankle arthrodesis for primary ankle osteoarthritis. Foot Ankle Spec 2023 16 20–27. (10.1177/1938640020987741) 33472419

[15] Anigian K, Ahn J, Wallace SB, et al. Comparison of short-term outcomes after total ankle replacement and ankle arthrodesis: an ACS-NSQIP database study. Foot Ankle Spec 2023 16 300–306. (10.1177/19386400211043363) 34713739

[16] Silvestre J, Morningstar JL, Hoch C, et al. Consumer preferences for total ankle arthroplasty versus ankle arthrodesis: a conjoint analysis of 1,410 US healthcare consumers. J Am Acad Orthop Surg 2025 33 e979–e987. (10.5435/JAAOS-D-25-00236) 40623294

[17] Encinas R, Kiriluk SH, Hardman J, et al. Clinical outcomes and safety profile for total ankle arthroplasty and ankle arthrodesis for symptomatic ankle arthritis: a systematic review. J Am Acad Orthop Surg 2025. (10.5435/JAAOS-D-25-00638) 40991861

[18] Anastasio AT, Walley KC, Kim BI, et al. Nationally representative trends in incidence of procedures done concomitantly with primary and revision total ankle from 2012 to 2020. Foot Ankle Spec 2025 18 269–278. (10.1177/19386400231216330) 38083850

[19] Comisi C, De Mauro D, Greco T, et al. Fixed-bearing versus mobile-bearing prostheses in total ankle arthroplasty: a systematic review and meta-analysis. J Clin Med 2025 14 6178. (10.3390/jcm14176178) 40943938 PMC12429794

[20] Robert SK, Chryssa N, Jun ML, et al. Comparison of patient reported outcomes using the Manchester-Oxford foot questionnaire and surgical complications following total ankle replacement in rheumatoid arthritis versus osteoarthritis. J Foot Ankle Surg 2025 64 791–795. (10.1053/j.jfas.2025.05.019) 40623496

